# Pinocembrin suppresses TGF-β1-induced epithelial-mesenchymal transition and metastasis of human Y-79 retinoblastoma cells through inactivating αvβ3 integrin/FAK/p38α signaling pathway

**DOI:** 10.1186/2045-3701-4-41

**Published:** 2014-08-12

**Authors:** Kun-Shiang Chen, Ming-Der Shi, Chi-Sheng Chien, Yuan-Wei Shih

**Affiliations:** 1 Department of Optometry, Chung Hwa University of Medical Technology, Tainan 71703, Taiwan; 2 Department of Medical Technology, Kaohsiung Veterans General Hospital Tainan Branch, Tainan 71051, Taiwan; 3 Department of Medical Laboratory Science and Biotechnology and Graduate Institute of Biological Technology, Chung Hwa University of Medical Technology, Tainan 71703, Taiwan; 4 Department of Orthopaedic Surgery, Chi Mei Medical Center, Tainan 71067, Taiwan; 5 Department of Food Nutrition, Chung Hwa University of Medical Technology, Tainan 71703, Taiwan; 6 Department of Biological Science and Technology and Graduate Institute of Biomedical Science, Chung Hwa University of Medical Technology, Tainan 71703, Taiwan

**Keywords:** Pinocembrin, TGF-β1, Invasion, Migration, αv and β3 integrin

## Abstract

**Background:**

Pinocembrin is the most abundant flavonoid in propolis. In this study, we investigated the antimetastatic effect of pinocembrin on TGF-β1-induced epithelial-mesenchymal transition (EMT) and metastasis of human Y-79 retinoblastoma cells.

**Results:**

Firstly, the results showed that pinocembrin significantly suppresses the TGF-β1-induced abilities of the invasion and migration of Y-79 cells under non-cytotoxic concentration. Pinocembrin decreased TGF-β1-induced expression of vimentin, N-cadherin, αv and β3 integrin in Y-79 cells. Molecular data also showed pinocembrin inhibits the activation of focal adhesion kinase (FAK) and p38α signal involved in the downregulation of enzyme activities, protein and messenger RNA levels of matrix metalloproteinase-2/9 (MMP-2/-9) induced by TGF-β1. Next, pinocembrin also strongly inhibited the degradation of inhibitor of kappaBα (IκBα) and the nuclear levels of nuclear factor kappa B (NF-κB). Also, a dose-dependent inhibition on the binding ability of NF-κB was further observed under pinocembrin treatment.

**Conclusions:**

Presented results indicated that pinocembrin inhibits TGF-β1-induced epithelial-mesenchymal transition (EMT) and metastasis of Y-79 cells by inactivating the αvβ3 integrin/FAK/p38α signaling pathway. Thus, our findings point to the anticancer potential of pinocembrin against retinoblastoma cells.

## Background

Retinoblastoma is the most common primary intraocular tumor in children (0–5 years). In the United States, this disease presents most frequently as unilateral sporadic tumors and less frequently as bilateral hereditary tumors. There are two types of this disease, the genetic-heritable type and non-genetic-non-heritable type. About 55 percent of children with retinoblastoma have the non-genetic type. Children with the inherited form have a high risk of developing other cancers later in life. If untreated, the patients die of intracranial extension and disease dissemination within two years [1]. Human retinoblastoma exhibits the patterns of invasion and metastasis. Direct invasive spreads along the optic nerve to the brain and can also seed the orbital tissue and adjacent bone [2]. Current treatments include enucleation, external beam radiotherapy, cryotherapy, photocoagulation photocoagulation, and chemotherapy [3]. Although these methods achieve survival rates of over 95%, there remains a need for better treatment alternatives to improve visual results and to avoid enucleation in hereditary retinoblastoma [4-6]. Thus, effective chemopreventive treatment for metastasis would have an important impact on retinoblastoma mortality rates.

The metastasis of tumor cells is a complex, multistage process. To facilitate the cell motility, invading cells need to change cell-cell adhesion properties, rearrange the extracellular matrix (ECM) environment, suppress anoikis and reorganize their cytoskeletons [7]. Integrins are a family of transmembrane adhesion receptors comprised of 19α and 8β subunits that interact non-covalently to form up to 24 different heterodimeric receptors. Integrin binds to ECM proteins or integrin cross-linking increases the tyrosine phosphorylation of FAK [8]. FAK is a nonreceptor tyrosine kinase primarily localized to cell-matrix adhesions which acts as a central regulator of focal adhesion influencing cell survival, differentiation, proliferation, migration and tissue remodeling [9-11]. Once localized to sites of transmembrane integrin receptor clustering, tyrosine-phosphorylated FAK plays an important role in signal transduction triggered by diverse extracellular signals [12] and represents a convergent point for synergistic interaction between signal pathways activated by growth factors and integrins [13-15]. Recently a conjunction between cancer stem cells and epithelial-mesenchymal transition (EMT) has been reported, and it has been shown that morphological and molecular changes occur when epithelial cells lose their characteristics, gain mesenchymal properties, and become motile, playing a critical role in the invasion of cancer cells [16,17]. The expression of Snail (Snail1), Slug (Snail2), ZEB1 or Twist, along with the expression of mesenchymal markers such as N-cadherin and vimentin, and the loss of E-cadherin are key molecular markers of EMT [18]. Transforming growth factor beta-1 (TGF-β1) has been identified as the main inducer of tumor EMT. The TGF-β1 is a pleiotropic cytokine that can mediate a wide spectrum of cellular effects through a variety of signaling pathways. Several studies have reported that TGF-β1 can promote angiogenesis, facilitate invasion, and suppress the host immune system [19].

**Figure 1:**
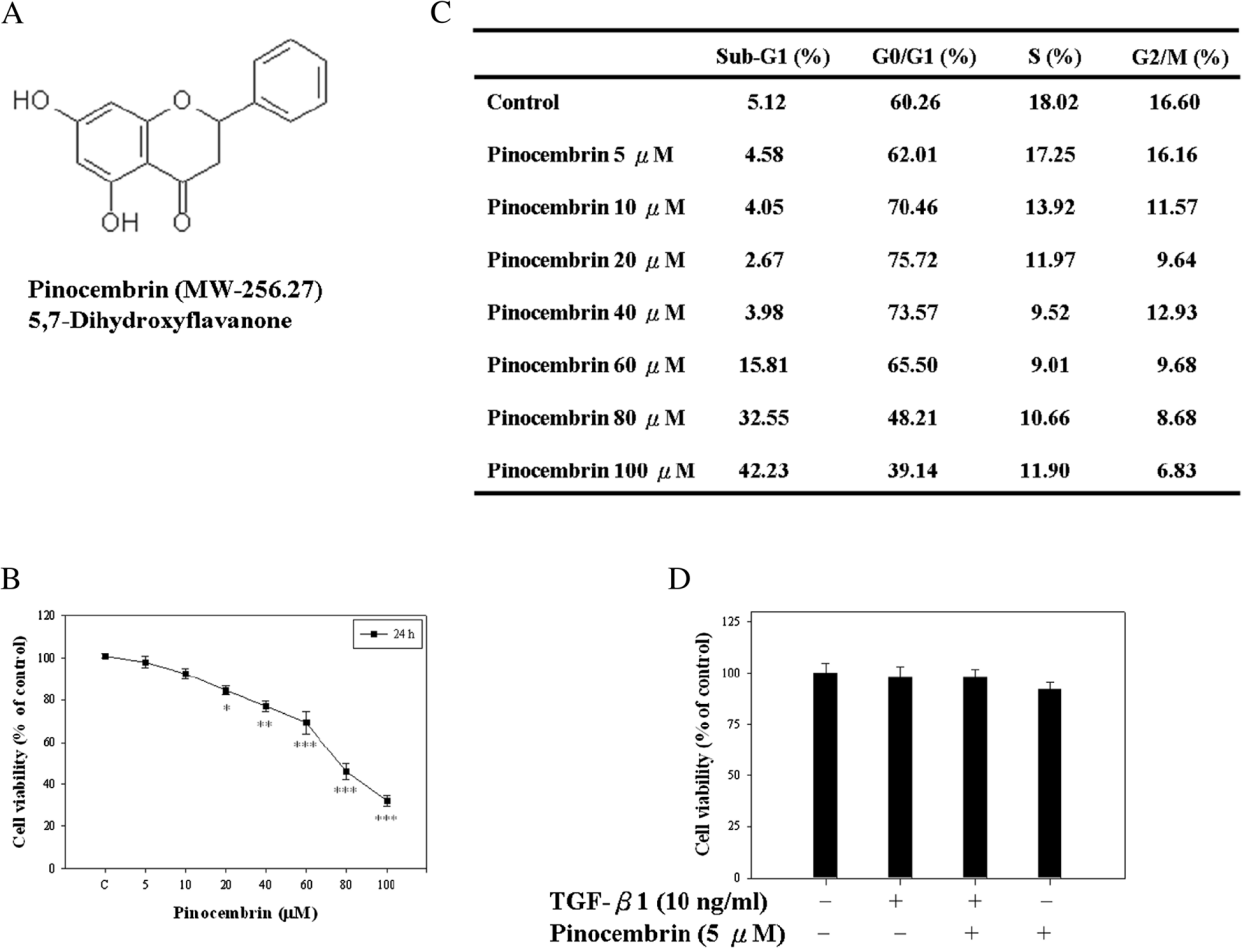
**The effect of pinocembrin on the viability and growth of Y-79 cells. (A)** The chemical structure of pinocembrin. **(B)** Cultured cells were treated with or without pinocembrin under various concentrations (0, 5, 10, 20, 40, 60, 80 and 100 μM) for 24 h. Thereafter, cell viability was determined by MTT assay. **(C)** The cells were fixed and stained with PI, and the cell cycle distribution was then analyzed by flow cytometry (FACS). **(D)** Cells were treated with or without of drugs (10 ng/ml TGF-β1 and 5 μM pinocembrin) for 24 h and the cell viability was determined by MTT assay. Values represent mean ± SD of three independent experiments (*p < 0.05, **p < 0.01, ***p < 0.001 compared with the untreated control (dose 0).

Pinocembrin (5,7-dihydroxyflavanone, Figure 1A) is a water insoluble flavonoid in propolis which is extracted as a pure compound. It is also found in nut pines, eucalyptus leaves and acacia gum. Now pinocembrin can be procured by synthesis and therefore is in abundance [20]. Pinocembrin has been shown to possess extensive pharmacological effects including antimicrobial [21,22], antioxidant [23], anti-mutagenic, and anti-inflammatory activities [24,25]. Although many studies have shown that pinocembrin possess various biological properties, the mechanisms by which this compound acts against cancers remain unclear. Based on our results, we hypothesized that pinocembrin might have antimetastatic property. Thus, this study utilized TGF-β1 as an inducer to induce the EMT of Y-79 retinoblastoma cells, and investigated the antimetastatic effect of pinocembrin on this cell model as well as its active mechanisms.

## Methods

### Reagents and antibodies

Pinocembrin (purity≧95%) was purchased from Extrasynthese (Genay, France). Poly-D-lysine, DMSO, Tris–HCl, EDTA, SDS, phenylmethylsulfonyl fluoride, bovine serum albumin (BSA), gelatin, crystal violet, leupeptin, Nonidet P-40, deoxycholic acid, sodium orthovanadate, mouse monoclonal antibody specific for αvβ3 integrin, and a selective αvβ3 integrin antagonist cyclic RGD (cycloRGDfV) peptide were purchased from Sigma-Aldrich (St. Louis, MO, USA); the protein assay kit was obtained from Bio-Rad Laboratories (Hercules, CA, USA). Dulbecco’s phosphate buffer solution (PBS), trypsin-EDTA, and RPMI 1640 medium were purchased from Life Technologies, Inc. (Gibco/BRL, Gaithersburg, MD). Matrigel was purchased from BD Biosciences (Bedford, MA). Antibodies against E-cadherin, N-cadherin, vimentin, FAK, FAK (Tyr397, Tyr576, Tyr925), ERK1/2, ERK1/2 (Thr202/Tyr204), JNK1/2, JNK1/2 (Thr183/Tyr185), p38α, and p38α (Thr180/Tyr182) were purchased from Cell Signaling Tech. (Beverly, MA, USA). NF-κB (p50 and p65), β-actin, and C23 antibodies were purchased from BD Transduction Laboratories (San Diego, CA, USA). An enhanced chemiluminescence (ECL) kit was purchased from Amersham GE Healthcare UK Ltd (Buckinghamshire, England).

### Cell culture and pinocembrin treatment

The human retinoblastoma Y-79 cell line was obtained from BCRC (Bioresource Collection and Research Center in Hsin-Chu, Taiwan). Cells were cultured at 37°C in a humidified atmosphere of 5% CO_2_-95% air. In medium supplemented with 10% fetal calf serum, 2 mM L-glutamine, 1 mM sodium pyruvate, and antibiotics (100 U/ml of penicillin and 100 mg/ml of streptomycin). Y-79 cells were grown in suspension at a concentration of 10^5^-10^6^ cells/ml. For all anti-metastatic examinations, cells were cultured in poly-D-lysine-coated culture plates. The stock solution of pinocembrin was dissolved in dimethyl sulfoxide (DMSO) and sterilized by filtration through 0.2 μm disc filters. Appropriate amounts of stock solution (1 mg/ml in DMSO) of pinocembrin were added to the cultured medium to achieve the indicated concentrations (the final DMSO concentration was less than 0.2%).

### Cell viability assay

To evaluate the cytotoxicity of pinocembrin, a MTT [3-(4,5-dimethylthiazol-2-yl)-2,5-diphenyl-tetrazoliumbromide] assay was performed to determine the cell viability [26]. Briefly, cells were seeded at a density of 4 × 10^4^ cells/ml in a 24-well plate for 24 h. Then, the cells were treated with or without pinocembrin under various concentrations for 24 h. Each concentration was repeated three times. Also, to further investigate whether pinocembrin and/or TGF-β1 influence cell viability, Y-79 cells were treated with the presence or absence of drugs (10 ng/ml TGF-β1 and 5 μM pinocembrin) for 24 h. After the exposure period, the medium was removed and followed by washing of cells with PBS. Then, the medium was changed and incubated with MTT solution (5 mg/ml)/well for 4 h. The medium was removed, and formazan was solubilized in isopropanol and measured spectrophotometrically at 563 nm. The percentage of viable cells was estimated by comparing with the untreated control cells.

### Flow cytometric assay

The effects of pinocembrin on Y-79 cell-cycle progression and TGF-β1-induced avβ3 integrin expression were determined by flow cytometry using FACScan (Becton Dickinson Immunocytometry Systems, UK). Firstly, to analyze the cell-cycle distribution, the cells were first treated with various concentrations of pinocembrin for 24 h, and then were collected by trypsinization, fixed in 75% absolute ethanol, washed in PBS, and resuspended in 1 ml of PBS containing 0.5 mg/ml RNase A and 0.01 mg/ml propidium iodide (PI) in the dark for 30 min at room temperature. The cell-cycle profiles were analyzed by a flow cytometer. The percentage of cells in the sub-G1, G0/G1, S, and G2/M phases of the cell cycle was analyzed by the ModFit LT 3.0 software (Verity Software, Topsham, ME). Furthermore, the cell surface expression of avβ3 integrin was determined using flow cytometry. Y-79 cells were plated in six-well dishes. The cells were then washed with PBS and detached with trypsin at 37°C. Cells were fixed for 10 min in PBS containing 1% paraformaldehyde. After rinsing in PBS, the cells were incubated with a mouse anti-human antibody against avβ3 integrin (1:100) for 1 h at 4°C. Cells were then washed again and incubated with fluorescein isothiocyanate-conjugated goat anti-rabbit secondary IgG (1:100; Leinco Tec. Inc., St. Louis, MO, USA) for 45 min and analyzed by flow cytometry.

### Cell-matrix adhesion assay

Y-79 cells were pretreated with 5 μg/ml anti-αvβ3 monoclonal antibody (mAb) or 80 nM cyclic RGD peptide (cyclo-RGDfV) for 30 min and then stimulated with 10 ng/ml TGF-β1 in the presence or absence of 5 μM pinocembrin for 24 h. Subsequently, cells were seeded at a density of 1 × 10^5^ cells/ml in a 24-well plate and coated with 500 μl type IV collagen (10 μg/ml); then they were cultured for 30 min. Then, non-adherent cells were removed by PBS washes, and adherent cells were fixed in ethanol. After staining with 0.1% crystal violet, fixed cells were lysed in 0.2% Triton X-100, and measured spectrophotometrically at 550 nm.

### Transwell invasion and migration assay

Invasion and migration assay were performed by using Hanging Cell Culture-inserts (BD Biosciences, San Jose, CA; pore size, 8-μm) in 6-well dishes. The ability of Y-79 cells to pass through filters coated with Matrigel (BD Biosciences) was measured by invasion assay. Matrigel was diluted to 200 μg/ml with filtered distill water and applied to the upper surface of the filter inserts. Briefly, Y-79 cells were pretreated with 5 μg/ml anti-αvβ3 monoclonal antibody (mAb) or 80 nM cyclic RGD peptide (cyclo-RGDfV) for 30 min and then stimulated with 10 ng/ml TGF-β1 in the presence or absence of 5 μM pinocembrin. After 24 h, cells were detached by trypsin and resuspended in serum-free medium. Medium containing 10% fetal bovine serum was applied to the lower chamber as a chemoattractant, and then cells were seeded on the upper filter at a density of 1 × 10^5^ cells/ml in the serum-free medium. The plates were incubated for 24 h at 37°C in 5% CO2, filter inserts were removed from the wells and the cells on the upper surface of the filter were wiped with a cotton-tipped swab. Filters were fixed with methanol for 10 min and stained with Giemsa dye for 1 h, and then the cells that had invaded the lower surface of the filter were counted under a light microscope. The data are presented as the average number of cells attached to the bottom surface from randomly chosen fields. Each experiment was carried out in triplicate. To measure the migration ability of Y-79 cells, cells were seeded into a transwell with 8 μm pore polycarbonate filters which were not coated with matrigel. Migrating cells were treated with the presence or absence of drugs (TGF-β1 and pinocembrin). Migration assay was measured as described in the invasion assay.

### Gelatin zymography assay

Cells (4 × 10^5^ cells/ml) were seeded into the culture and stimulated with 10 ng/ml TGF-β1 for 2 h and then incubated in different concentrations of pinocembrin (0, 1, 2.5, and 5 μM) for 24 h. Subsequently, the conditioned medium was collected and gelatin zymography was performed to examine the activities of MMP-2 and MMP-9. Samples were mixed with loading buffer and electrophoresed on 8% SDS-polyacrylamide gel containing 0.1% gelatin. Electrophoresis was performed at 140 and 110 V for 3 h. Gels were then washed twice in zymography washing buffer (2.5% Triton X-100 in double-distilled H_2_O) at room temperature to remove SDS, followed by incubation at 37°C for 12–16 h in zymography reaction buffer (40 mM Tris–HCl, 10 mM CaCl_2_, 0.02% NaN_3_), stained with Coomassie blue R-250 (0.125% Coomassie brilliant blue R-250, 0.1% amino black, 50% methanol, 10% acetic acid) for 1 h and destained with destaining solution (20% methanol, 10% acetic acid, 70% double-distilled H_2_O). Nonstaining bands representing the levels of the latent forms of MMP-2 and MMP-9 were quantified by densitometer measurement using a digital imaging analysis system.

### Isolation of total RNA, reverse transcriptase polymerase chain reaction (RT-PCR) and DNA electrophoresis

Total RNA was isolated from human Y-79 cells using the total RNA Extraction Midiprep System (Viogene BioTek Corporation, Taiwan). Total RNA (2 μg) was transcribed to 20 μl cDNA with 1 μl dNTPs (2.5 mM), 1 μl oligo dT (10 pmole/μl), 1 μl RTase (200 U), 1 μl RNase inhibitor and 5 X reaction buffer. The appropriate primers (sense of MMP-2, 5′-GGCCCTGTCACTCCTGAGAT-3′ nt 1337–1356; antisense of MMP-2, 5′-GGCATCCGGTTATCGGGGA-3′, nt 2026–2007; sense of MMP-9, 5′-AGGCCTCTACAGAGTCTTTG-3′ nt 1201–1220; antisense of MMP-9, 5′-CAGTCCAACAAGAAAGGACG-3′, nt 1700–1683; sense of GADPH, 5′-CGGAGTCAACGGATTGGTGTT-3′, nt 94–126; antisense of 5′-AGCCTTCTCCATGGTTGGTGAAGAC-3′, nt 399–375) were used for PCR amplifications. PCR was performed with Platinum Taq polymerase (Invitrogen) under the following conditions: 30 cycles of 94°C for 1 min, 59°C (MMP-2) or 60°C (MMP-9 and GAPDH) for 1 min, 72°C for 1 min followed by 10 min at 72°C.

### Western blotting assay

The preparation of cytosolic and nuclear fractions of the cells was performed as described previously [27]. Western blotting was performed as follows. The denatured samples (50 μg purified protein) were resolved on 10-12% SDS-PAGE gels. The proteins were then transferred onto nitrocellulose membranes. Non-specific binding of the membranes was blocked with Tris-buffered saline (TBS) containing 1% (w/v) nonfat dry milk and 0.1% (v/v) Tween-20 (TBST) for more than 2 h. Membranes were washed with TBST three times for 10 min and incubated with an appropriate dilution of specific primary antibodies in TBST overnight at 4°C. Subsequently, membranes were washed with TBST and incubated with an appropriate secondary antibody (horseradish peroxidase-conjugated goat antimouse or antirabbit IgG) for 1 h. After washing the membrane three times for 10 min in TBST, the bands detection was carried out by enhanced chemiluminescence using ECL Western blotting detection reagents and exposed ECL hyperfilm in FUJFILM Las-3000 mini (Tokyo, Japan). Then proteins were quantitatively determined by densitometry using FUJFILM-Multi Gauge V3.0 software.

### Electrophoretic mobility shift assay (EMSA)

Cell nuclear proteins were extracted with a nuclear extract buffer and measured by electrophoretic mobility shift assay (EMSA) [28]. Cells (1 × 10^5^/ml) were collected in PBS buffer (pH 7.4) and centrifuged at 2000 × *g* for 5 min at 4°C. Cells were lysed with buffer A (10 mM HEPES, 1.5 mM MgCl_2_, 10 mM KCl, 0.5 mM DTT, and 0.5 mM PMSF (pH 7.9) containing 5% NP-40) for 10 min on ice, and this was followed by vortexing to shear the cytoplasmic membranes. The lysates were centrifuged at 2000 × *g* for 10 min at 4°C. The pellet containing the nuclei was extracted with high salt buffer B (20 mM HEPES, 420 mM NaCl, 1.5 mM MgCl_2_, 0.5 mM DTT, 0.5 mM PMSF, 0.2 mM EDTA, and 25% glycerol) for 15 min on ice. The lysates were centrifugated at 13000 × *g* for 10 min at 4°C. The supernatant containing the nuclear proteins was collected and frozen at -80°C until use. The protein content of nuclear fractions was determined with Bio-Rad protein assay. Synthetic double-strand oligonucleotides of the consensus NF-κB binding sequence, 5′-AGTTGAGG GGACTTTCCCAGGC-3′ and 3′-TCAACTCCCCTGAAAGGGTCCG-5′, were 5′end- labeled with biotin. Binding reactions containing 5 μg of nuclear proteins, double-distilled H_2_O, 2 μl 10-fold binding buffer, 2 μg poly (dI · dC) and 2 pmol oligonucleotide probe were incubated for 15 min at room temperature. Specific competition binding assays were performed by adding a 200-fold excess of an unlabeled probe as a specific competitor. Following formation of protein-DNA complexes, samples were loaded on a 6% nondenaturing polyacrylamide gel in 0.5 × TBE buffer and then transferred to positively charged nitrocellulose membranes (Millipore, Bedford, MA, USA) by a transfer blotting apparatus and cross-linked in a Stratagene crosslinker. Gel shifts were visualized with streptavidin-horseradish peroxidase followed by chemiluminescent detection.

### Transient transfection and luciferase report gene assays

NF-κB transcriptional activity was measured by NF-κB-luciferase report gene expression. Y-79 cells (4 × 10^4^ cells/well) were plated in six-well plates. The cells were transiently co-transfected with the plasmids, pGL3-NF-κB, pCMV-β-gal and pcDNA3.1 using Lipofectamine Plus according to the manufacturer’s protocol. Briefly, a transfection mixture containing 0.5 μg pGL3-NF-κB and 0.2 μg pCMV-β-gal was mixed with the Lipofectamine Plus reagent and added to the cells. After 8 h, the cells were stimulated with 10 ng/ml TGF-β1 for 2 h and then incubated in 5 μM pinocembrin for 0, 1, 3, 6, and 9 h. Then the NF-κB-luciferase activity was measured according to the manufacturer’s instructions (Promega). Briefly, cells were washed twice with cold PBS and lysed by adding 100 μl 1X reporter lysis buffer (24 mM Tris–HCl (pH 7.8), 2 mM dithiotreitol, 2 mM EDTA, 10% glycerol, and 1% Triton X-100) (Promega, Madison, WI, USA). After centrifugation (13000 × *g*, 2 min), samples were measured for luciferase activity by using a Sirus Luminometer (Berthold Detection System; OAK Ridge, TN) using 10 μl of cell lysate and 100 μl of luciferase assay reagent (Promega). Luciferase activity was measured with a 10 s delay and 30 s integration time and was normalized to β-galactosidase or Renilla luciferase activity to determine transfection efficiency. The values shown represent an average of three independent transfections and each transfection was carried out in triplicate.

### Statistical analysis

Data were expressed as means ± standard deviation of three independent experiments. Statistical comparisons of the results were made using analysis of variance (ANOVA). Significant differences were established at *p* ≤ 0.05.

## Results

### Cytotoxicity of pinocembrin to Y-79 cells

In this study, we first examined the effect of pinocembrin on cell cytotoxicity in Y-79 cells. As shown in Figure 1B, pinocembrin did not affect the cell cytotoxicity of Y-79 cells at concentrations ranging from 0–5 μM. In order to verify this, the dose range was applied in all subsequent experiments to avoid the influence of cell growth and cytotoxicity on the observed papameters, and a series of studies were performed to measure the cell growth and cytotoxicity upon pinocembrin stimulation. Firstly, the effects of pinocembrin on Y-79 cell-cycle progression were measured by flow cytometry (Figure 1C) which revealed that a treatment with 5 μM pinocembrin had no effect on the cell-cycle distribution for 24 h. At higher concentrations (10, 20, 40, 60, 80 and 100 μM), pinocembrin increased the G1 fraction (from 60.26% to 73.57%), and caused an apparent accumulation of the cells in the sub-G1 phase, while decreasing the S fraction in a dose-dependent manner. Therefore, the results demonstrated that a 24 h treatment of pinocembrin at a concentration ranging from 0 to 5 μM had no cytotoxicity to Y-79 cells. Also, as illustrated in Figure 1D, in the presence of TGF-β1, pinocembrin at a concentration of 5 μM did not have any toxic effects on cell viability. Thus, non-cytotoxic concentrations of pinocembrin (0–5 μM) were used in subsequent experiments.

### Pinocembrin suppresses TGF-β1-induced cell-matrix adhesion, invasion, migration, and epithelial- mesenchymal transition in Y-79 cells via αvβ3 integrin

**Figure 2:**
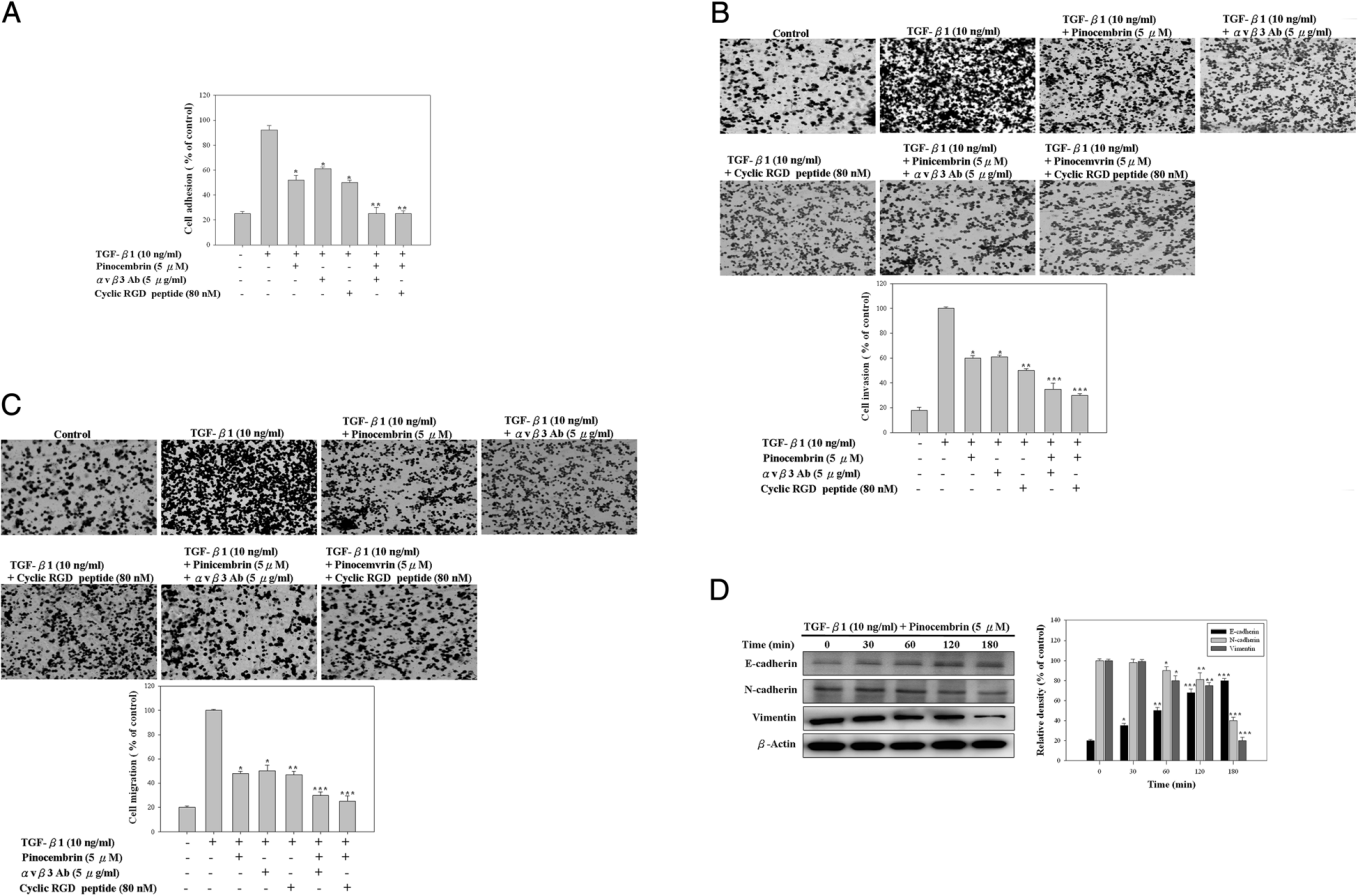
**The inhibitory effect of pinocembrin on TGF-β1-induced cell-matrix adhesion, invasion, migration, and epithelial-mesenchymal transition of Y-79 cells involves activation of αvβ3 integrin. (A)** Y-79 cells were pretreated with 5 μg/ml anti-αvβ3 monoclonal antibody (mAb) or 80 nM cyclic RGD peptide (cyclo-RGDfV) for 30 min and then stimulated with 10 ng/ml TGF-β1 in the presence or absence of 5 μM pinocembrin for 24 h, and were then subjected to analyses for cell-matrix adhesion as described in Methods. **(B)** In invasion assay, cells were pretreated with 5 μg/ml anti-αvβ3 monoclonal antibody (mAb) or 80 nM cyclic RGD peptide (cyclo-RGDfV) for 30 min and then stimulated with 10 ng/ml TGF-β1 in the presence or absence of 5 μM pinocembrin for 24 h. Then invasion assay were measured by transwell for 8 h; polycarbonate filters (pore size, 8 μm) were precoated with matrigel. After 8 h incubation, cells on the bottom side of filter were fixed, stained, and counted. **(C)** In migration assay were measured by transwell for 6 h with polycarbonate filters. After 6 h incubation, cells on the bottom side of filter were fixed, stained, and counted. **(D)** Cells were pre-treated with TGF-β1 (10 ng/ml) in the absence or presence of 5 μM pinocembrin for 0, 30, 60, 120, 180 min, and then used to the cell-lysate extracts were measured by Western blotting with anti-E-cadherin, anti-N-cadherin, anti-vimentin, and anti-β-actin antibodies as described in Methods. Values represent mean ± SD of three independent experiments (*p < 0.05, **p < 0.01, ***p < 0.001 compared with the TGF-β1 treatment only).

Previous studies have shown that TGF-β1 affects cells invasion and migration through αvβ3 integrin signaling [29]. Therefore, we hypothesized that the αvβ3 integrin signaling pathway may be involved in TGF-β1-induced cell-matrix adhesion, invasion, and migration of Y-79 cells. Firstly, to investigate whether low-cytotoxic pinocembrin inhibits the TGF-β1-induced adhesive properties of Y-79 cells, a cell-matrix adhesion assay was examined. As shown in Figure 2A, treatment of Y-79 cells with TGF-β1 (10 ng/ml) induced cell adhesion. In particular, 5 μM pinocembrin inhibited the TGF-β1-induced cell adhesion of Y-79 cells by 47.3%. Y-79 cells were pretreated with 5 μg/ml anti-αvβ3 monoclonal antibody (mAb) or 80 nM cyclic RGD peptide (cyclo-RGDfV) for 30 min and then stimulated with 50 ng/ml TGF-β1 in the presence or absence of 5 μM pinocembrin for 24 h. The pretreatment of Y-79 cells with anti-αvβ3 monoclonal antibody or cyclic RGD peptide decreased the TGF-β1-induced cell adhesion by 39.7% or 49.3%, respectively. Also, the combining treatment with anti-αvβ3 monoclonal antibody or cyclic RGD peptide and pinocembrin reduced TGF-β1-induced cell adhesion by 75% or 75.7%, respectively, as compared with that of TGF-β1 treatment only. The cyclic RGD peptide (cyclo-RGDfV) has been demonstrated to bind αvβ3 at high affinity and block its function effectively at low concentrations. Furthermore, we examined the effect of pinocembrin on Y-79 cell invasion and migration. As shown in Figure 2B and C, the cells were treated with 5 μM pinocembrin, the results showed that the cell invasion and migration of TGF-β1-induced Y-79 cells was significantly decreased, compared to the TGF-β1 treatment only. Pretreatment of cells for 30 min with 5 μg/ml anti-αvβ3 monoclonal antibody or 80 nM cyclic RGD peptide all markedly inhibited TGF-β1-induced cell invasion and migration. Also, the combining treatment with anti-αvβ3 monoclonal antibody or cyclic RGD peptide and pinocembrin reduced TGF-β1-induced cell invasion and migration by 64.3% or 70.3% and 65.7% or 74.0%, respectively, as compared with that of TGF-β1 treatment only. Epithelial-mesenchymal transition (EMT) is a crucial progression in the development of invasive cancer cells. We assessed the effect of pinocembrin on EMT markers. Pinocembrin treatment increased E-cadherin levels and decreased vimentin and N-cadherin by TGF-β1 stimulation (Figure 2D). Taken together, these results suggest that inhibition of TGF-β1-induced cancer adhesion, invasion, and migration of highly metastatic Y-79 cells by pinocembrin may occur via activation of the αvβ3 integrin receptor.

### Pinocembrin suppresses TGF-β1-induced integrin levels in Y-79 cells

**Figure 3:**
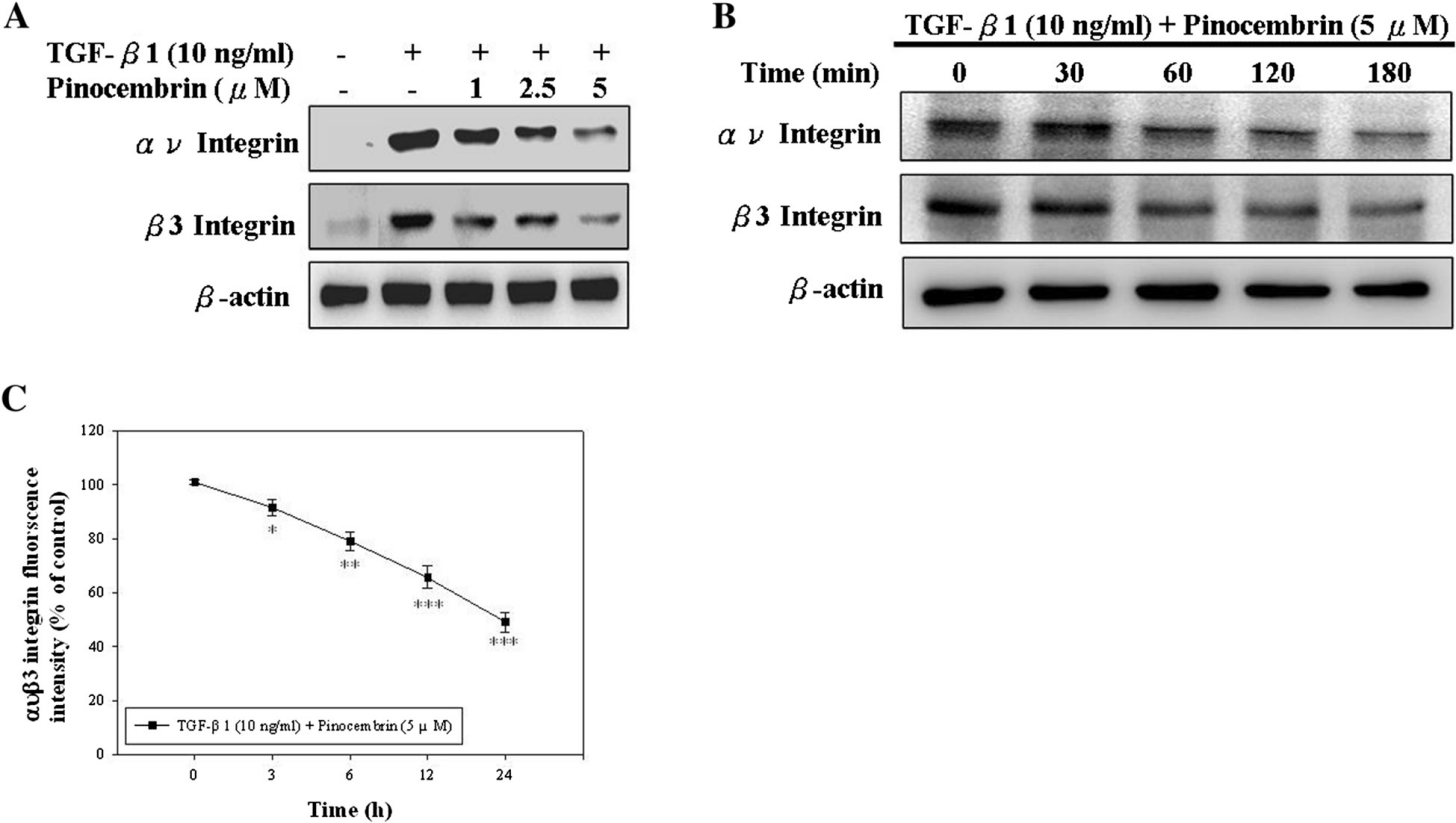
**The inhibitory effect of pinocembrin on TGF-β1-induced integrin levels in Y-79 cells. (A)** Cells were pretreated with TGF-β1 (10 ng/ml) and then incubated in the different concentrations of pinocembrin (0, 1, 2.5, and 5 μM) for 6 h, and the integrins were analyzed. **(B)** Cells were pretreated with TGF-β1 (10 ng/ml) and then incubated in 5 μM pinocembrin for different lengths of time (0, 30, 60, 120, and 180 mins), and then the integrins were analyzed. **(C)** The expressions of integrins were determined by flowcytometry. Values are expressed as mean ± SD of three independent experiments. *p < 0.05, **p < 0.01, ***p < 0.001 compared with the 0 h-treated time.

Integrins link the ECM to intracellular cytoskeletal structures and signaling molecules and are implicated in the regulation of a number of cellular processes, including adhesion, signaling, motility, survival, gene expression, growth, and differentiation [30]. Here we explored the effect of pinocembrin on TGF-β1-induced integrin expressions in Y-79 cells. Firstly, cells were pre-treated with TGF-β1 (10 ng/ml) and then incubated in different concentrations of pinocembrin (0, 1, 2.5, and 5 μM) for 6 h. As shown in Figure 3A, pinocembrin significantly inhibited the TGF-β1-induced expression levels of αν and β3 integrin in Y-79 cells. Furthermore, cells were pre-treated with TGF-β1 (10 ng/ml) and then incubated in 5 μM pinocembrin for different lengths of time (0, 30, 60, 120, and 180 mins). The subsequent time course experiment showed that the reduction of αν and β3 integrin expressions by pinocembrin also occurred in a time-dependent manner when pinocembrin was at 5 μΜ (Figure 3B). Additionally, flow cytometry data also showed that pinocembrin decreased the TGF-β1-induced the expression of ανβ3 integrin (Figure 3C).

### Pinocembrin suppresses TGF-β1-induced expressions of MMP-2 and MMP-9 in Y-79 cells

**Figure 4:**
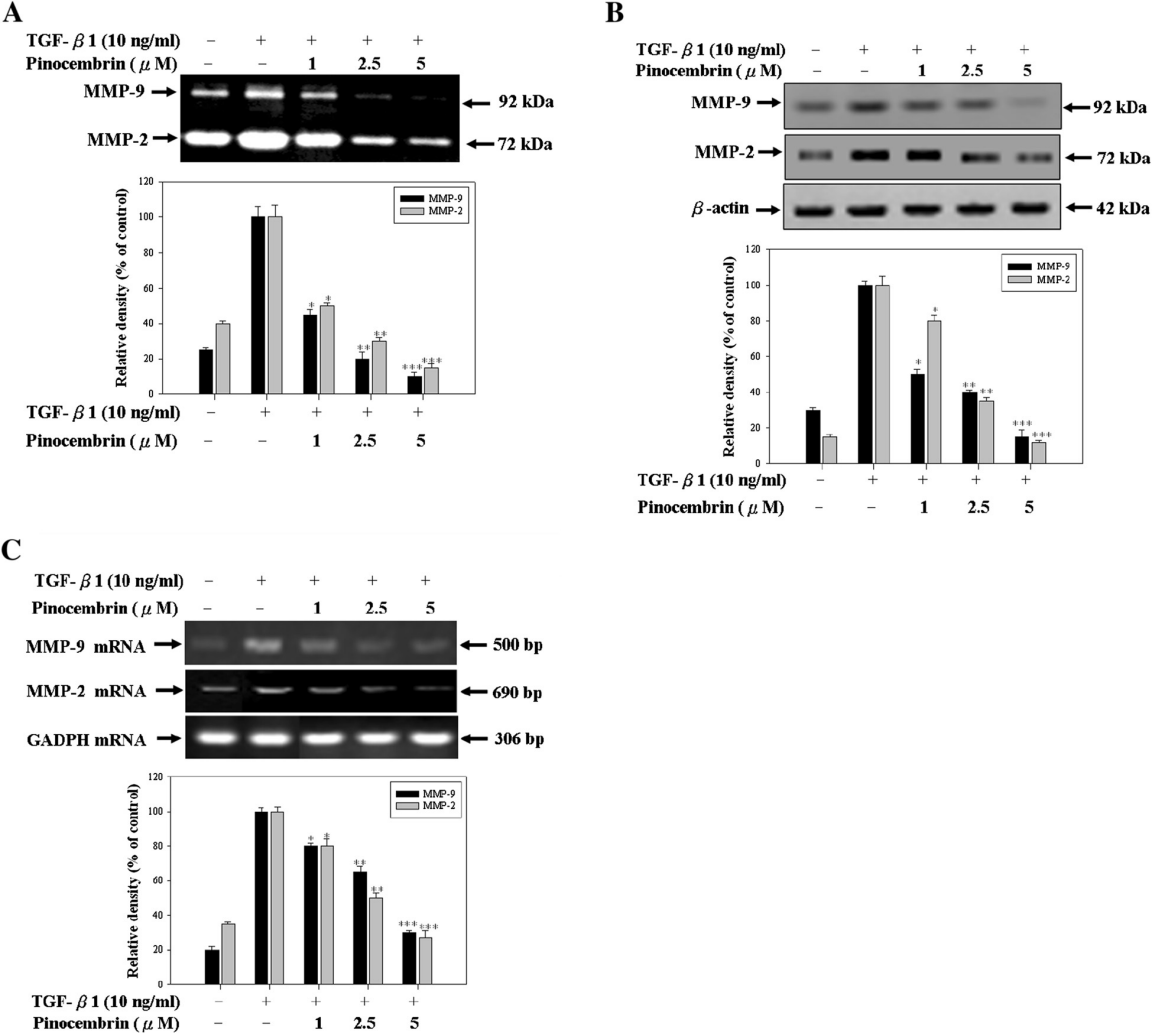
**The inhibitory effect of pinocembrin on TGF-β1-induced activities and expressions of MMP-2/-9 in Y-79 cells.** The cultured cells in serum-free medium were pretreated with TGF-β1 (10 ng/ml) and then incubated in the different concentrations of pinocembrin (0, 1, 2.5, and 5 μM). **(A)** The conditioned media were collected and MMP-2/-9 activities were determined by gelatin zymography. **(B)** The MMP-2/-9 protein expressions were analyzed by Western blotting. **(C)** The MMP-2/-9 mRNA expressions were analyzed by RT-PCR. β-Actin and GADPH was used as internal controls.

To investigate the inhibitory effect of pinocembrin on TGF-β1-induced MMP-2 and MMP-9 enzyme activities, gelatin zymography was performed to examine the activities of MMP-2 and MMP-9 using conditioned medium, which was collected and measured after TGF-β1 stimulation with or without pinocembrin treatment. Both MMP-2 and MMP-9 were detected in the conditioned media of Y-79 cells, while TGF-β1 significantly increased the MMP-2 and MMP-9 activities. Also, pinocembrin inhibited the MMP-2 and MMP-9 activities stimulated by TGF-β1 in a dose-dependent manner (Figure 4A). The zymography result obtained was further confirmed by Western blotting analysis. As shown in Figure 4B, treatment with 1 μM pinocembrin reduced the protein expression levels of MMP-2 or MMP-9 induced by TGF-β1, while 5 μM pinocembrin significantly inhibited the TGF-β1-induced protein expression levels of MMP-2 or MMP-9. To determine whether the inhibition of MMP-9 or MMP-2 protein expressions by pinocembrin was due to a decreased level of transcription, we performed RT-PCR and observed mRNA expressions of MMP-2 and MMP-9. As shown in Figure 4C, pinocembirn reduces the TGF-β1-induced MMP-2 and MMP-9 mRNA expressions of Y-79 cells in a dose-dependent manner. The data suggest pinocembrin prevents the transcription of MMP-2 and MMP-9 in response to TGF-β1. These results suggested that the anti-metastatic effect of pinocembrin is related to the inhibition of the enzymatically degradative processes of tumor metastasis.

### Pinocembrin inhibits TGF-β1-induced phosphorylation of FAK/p38α in Y-79 cells

**Figure 5:**
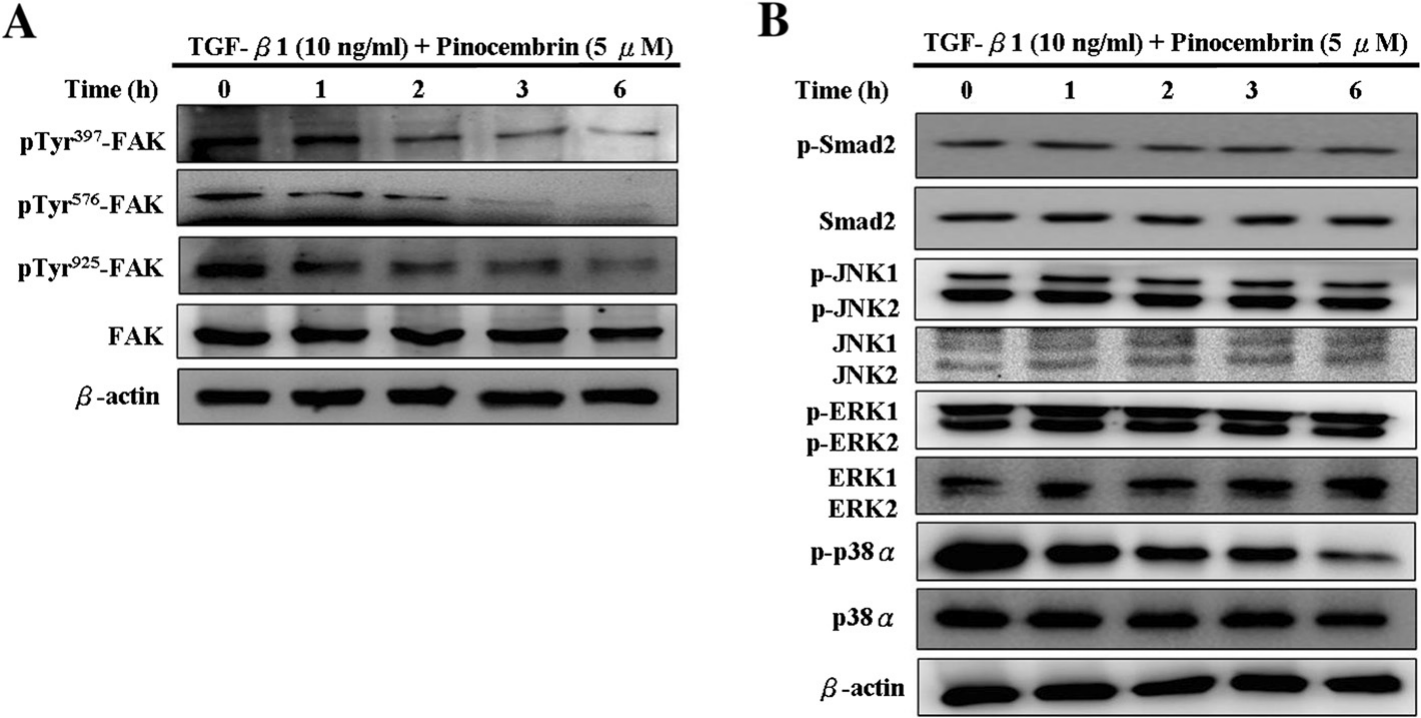
**The inhibitory effect of pinocembrin on TGF-β1-induced phosphorylation of FAK/p38α in Y-79 cells. (A and B)** Cells were pretreated with TGF-β1 (10 ng/ml) for 2 h and then incubated in 5 *μ*M pinocembrin for 0, 1, 2, 3, and 6 h. The total protein and phosphorylation levels of FAK (Tyr397, Tyr576, Tyr925), Smad2, JNK1/2, ERK1/2, p38α were measured by Western blotting. β-Actin was used as an internal control.

In many signaling milieus, the FAK signaling during adhesion has been shown to be capable of regulating integrin-mediated signaling and involved in cell metastasis, but the mechanism underlying its activity is only partly understood. To investigate proteins whether could potentially phosphorylate, we performed a time-course assay of the inhibitory effect of pinocembrin on the expressions of non-Smad (FAK/MAPK) and Smad signaling pathways by TGF-β1-induced to clarify the underlying mechanisms. As shown in Figure 5A, pinocembrin decreased the TGF-β1-induced the phosphorylation of FAK at the Tyr 397, 576, and 925 sites in Y-79 cells. However, pinocembrin did not cause any change in the total protein levels of total FAK. In addition, the MAPK family is also a target of FAK signaling, and hence we next investigated the phosphorylation of JNK, p38α and ERK1/2 in Y-79 cells. The data showed that pinocembrin decreased the TGF-β1-induced the phosphorylation of p38α, but not phospho-JNK and phospho-ERK in Y-79 cells. Moreover, we found that pinocembrin was unable to cause any significant deviation in the ratio of phospho-Smad2 to total Smad2 at any measure time (Figure 5B). Total protein levels of JNK, ERK, p38α and Smd2 did not change with TGF-β1 and pinocembrin treatment.

### Pinocembrin inhibits TGF-β1-induced DNA binding activity of NF-κB and the expressions of NF-κB and IκBα in Y-79 cells

**Figure 6:**
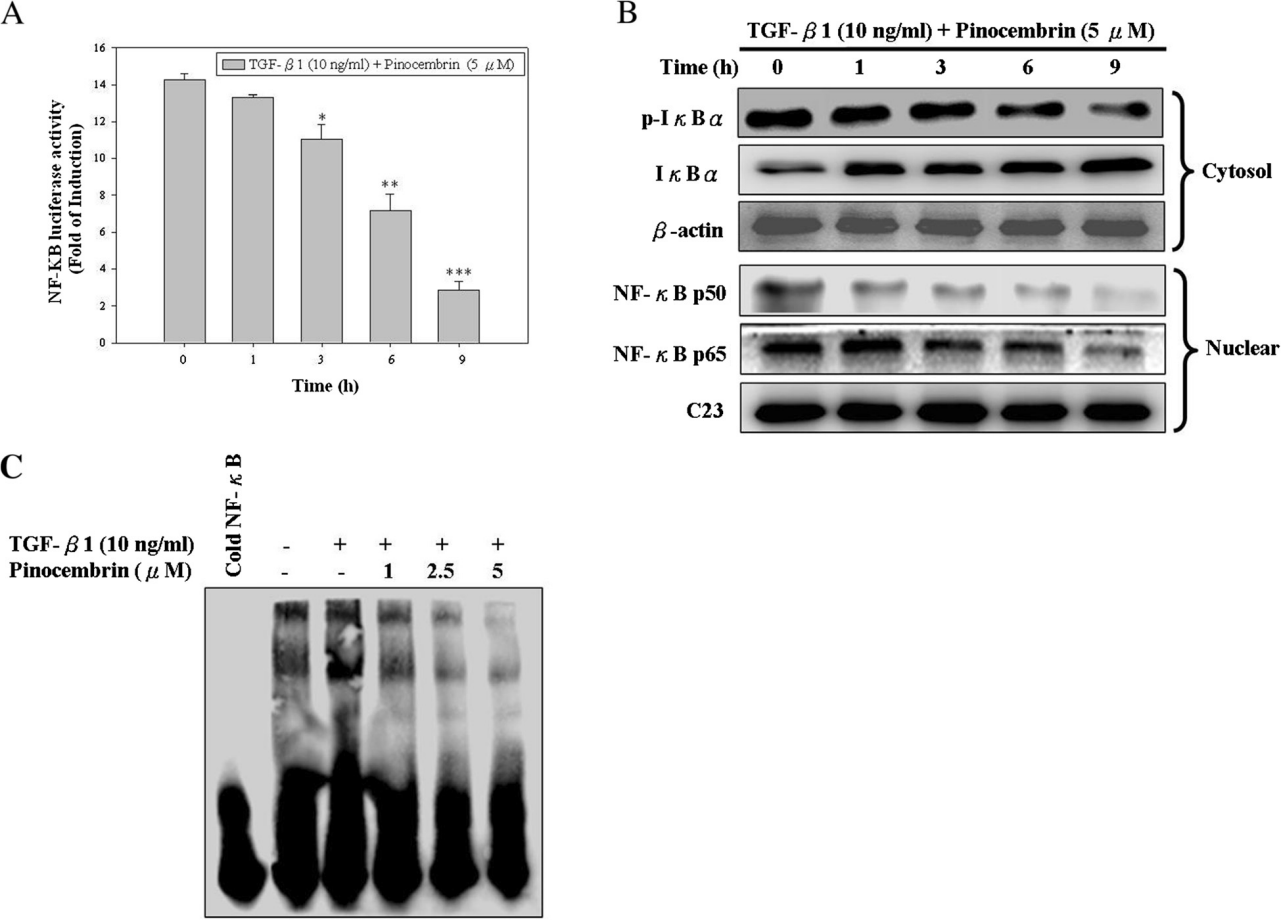
**The inhibitory effect of pinocembrin on TGF-β1-induced NF-κB transcriptional activity/expressions of NF-κB/IκBα phosphorylation and degradation in Y-79 cells. (A)** Cells were pretreated with TGF-β1 (10 ng/ml) for 2 h and then treated with 5 *μ*M pinocembrin for 0, 1, 2, 3, and 9 h, and the NF-κB transcriptional activity by luciferase report gene assays, as described in Methods. **(B)** Nuclear extracts were subjected to SDS–PAGE followed by Western blotting with specific antobodies (anti-NF-κB p50, anti-NF-κB p65, anti-p-IκBα, anti-IκBα). C23 and β-actin were used as internal control. **(C)** NF-κB DNA-binding activity by EMSA, as described in Methods. Lane 1: nuclear extracts incubated with 100-fold excess unlabeled consensus oligonucleotide (comp.) to confirm the binding specificity. Lane 2 represents nuclear extract from Y-79 cells in the absence of TGF-β1 (negative control). Values represent mean ± SD of three independent experiments. (*p < 0.05, **p < 0.01, ***p < 0.001 compared with the TGF-β1 treatment only).

The expressions of MMP-2 and MMP-9 genes are regulated through the transcriptional level interaction of NF-κB with their binding sequences in MMP-2 and MMP-9 gene promoters. NF-κB, one of the main molecular targets of chemopreventive phytochemicals [31], is a transcription factor involved in multiple cellular processes, including cytokine gene expression, cellular adhesion, apoptosis, and metastasis [32]. These inflammatory metastatic mediators were found to be altered by pinocembrin treatment. Therefore, we used transcriptional activation assays to determine whether pinocembrin affects NF-κB dependent transcription. Stimulation with TGF-β1 resulted in an approximately 5-fold increase in luciferase activity, and the TGF-β1-induced NF-κB activity was inhibited by pinocembrin treatment in a time-dependent fashion (Figure 6A). The p65 and p50 subunits of NF-κB have been demonstrated to exert critical activity in the transcription of MMP-2/MMP-9 genes. Moreover, the activation of NF-κB occurs through the phosphorylation of IκBα releasing the NF-κB for nuclear translocation and thus binding to the promoter sites of target genes. Therefore, we also performed a Western blot assay to detect expressions of NF-κB (p65 and p50) and IκBα to clarify the inhibitory action of pinocembrin. As shown in Figure 6B, pretreatment of the cells with 5 μM pinocembrin for 9 h significantly inhibited both IκBα degradation and NF-κB (p50/p65) nuclear translocation. Because TGF-β1-stimulated activation of NF-κB is correlated with IκBα degradation, pinocembrin blocked TGF-β1-induced IκBα degradation by inhibiting phosphorylation of IκBα. To further determine the possibility of NF-κB binding to MMP-2/MMP-9 promoter binding sites, EMSA was done with specific oligonucleotides As shown in Figure 6C, the TGF-β1-stimulated NF-κB DNA binding activities were strongly inhibited by pinocembrin at the concentration of 5 μM.

## Discussion

Retinoblastoma is the most common primary intraocular tumor in children (0–5 years). It is associated with generally poor prognosis due to its tendency toward local invasion and subsequent metastasis. Recently, antimetastatic agents have been defined as a new class of cancer chemopreventive agent. Pinocembrin is one of the flavonoids with the highest concentration levels in propolis. Flavonoids are a vast group of heterogeneous polyphenols that are thought to have positive effects on human health, including cancer prevention. Various studies have shown that pinocembrin exerts pleiotropic anticancer effects, including preventive, anticarcinogenic, and antiproliferative effects in various *in vivo* and *in vitro* models. In particular, pinocembrin could influence several processes and play important roles in the regulation of various molecular targets, including NF-κB [33], Smad2 and PPARγ transcription factors [34], Vascular endothelial growth factors [35], TGF-β1 [34], necrosis factor-alpha, interleukin-1beta, intercellular adhesion molecule-1, vascular cell adhesion molecule-1 inflammatory cytokines [36], p38 MAPK [37], ERK1/2 and PI3K/Akt protein kinases [38] and other enzymes (COX-2 and MMPs) [39,36].

Tumor promoting activity of TGF-β1 associated with the induction of EMT has been documented for different tumor types [40]. Several reports have shown that TGF-β1 has been identified as the main inducer of tumor EMT. The EMT induced by TGF-β1 results in the disruption of the polarized morphology of epithelial cells, formation of actin stress fibers, and enhancement of cell migration [41]. The TGF-β1 family of secreted factors is involved in controlling different biological processes, including cell proliferation, differentiation, and apoptosis. Furthermore, conjunction between TGF-β1 signaling and carcinoma cells in cell invasiveness and tumor metastasis has recently been reported [42]. These effects of TGF-β1 are related to its ability to induce EMT and stimulate cell migration. Furthermore, several non-Smad pathways have also been shown to mediate the cellular effects of TGF-β1. These include ERK, JNK, p38 MAPK, and PI3K/Akt. In this study, we have demonstrated that pinocembrin suppresses TGF-β1-induced EMT via the p38α signaling pathway.

We further examined the effect of the expression levels of E-cadherin, N-cadherin, and vimentin. E-cadherin, an adherent junction protein and type I transmembrane glycoprotein, plays a pivotal role in the maintenance of normal tissue architecture and the suppression of cancer invasion [43]. A previous study demonstrated that the loss of E-cadherin-mediated adhesion plays an important role in the transition of epithelial tumors from a benign to an invasive state [44]. More recent evidence indicates that the gain of expression of another adhesion molecule, N-cadherin, is associated with heightened invasive potential in tumor cells. N-cadherin has also been reported to induce a mesenchymal-scattered phenotype associated with reduced E level in a squamous cell carcinoma cell line [45]. Vimentin, a mesenchymal marker, has been was observed in a few cancer cells. It has previously been demonstrated that vimentin expression is substantially increased in cancer cells of hepatic metastasis [46]. Taken together, our results are consistent with the notion that the changes were associated with an increase in E-cadherin expression and a reduction in N-cadherin and vimentin in TGF-β1-treated Y-79 cells. Loss of cell-cell adhesion followed by the dissociation of epithelial structures is a prerequisite for increased cell motility and tumor invasion. These findings raise the possibility that N-cadherin and vimentin contribute directly to the invasive phenotype. In addition to promoting motile and invasive activities of Y-79 cells in vitro, the expressions of N-cadherin and vimentin greatly sensitized Y-79 cells to TGF-β1, stimulating marked increases in cell migration and invasion as well as in MMP-2/-9 production. When Y-79 cells were treated with pinocembrin, there dramatically reduced the activities and expressions of MMP-2/-9. It is of importance to note that MMP-2/-9 plays roles in tumor progression, as over-expression of the MMP-2/-9 gene in transgenic mice led to enhanced tumorigenesis in a breast cancer model [47]. Also, Sasaki et al. [48] reported that the survival rates of lung cancer patients were poor for those patients with an elevated MMP-2/-9 mRNA expression on the tumor tissue. We have demonstrated that reduction of proteolytically active MMP-2/-9 is involved in pinocembrin mediated-cell invasion and migration. In addition, the transcription of the MMP-2/-9 gene is regulated by upstream sequences, including motifs corresponding to the NF-κB binding element [49,50]. Here, we also found that pinocembrin inhibits MMP-2/-9 expression through preventing IκBα being phosphorylated and enhancing IκBα protein expression, both leading to inactivation of NF-kB DNA binding activity. A comprehensive analysis of MMP-2/-9 expression in Y-79 cells will be necessary to fully understand the biological properties of Y-79 cells and to promote the development of new therapeutic strategies.

## Conclusions

**Figure 7:**
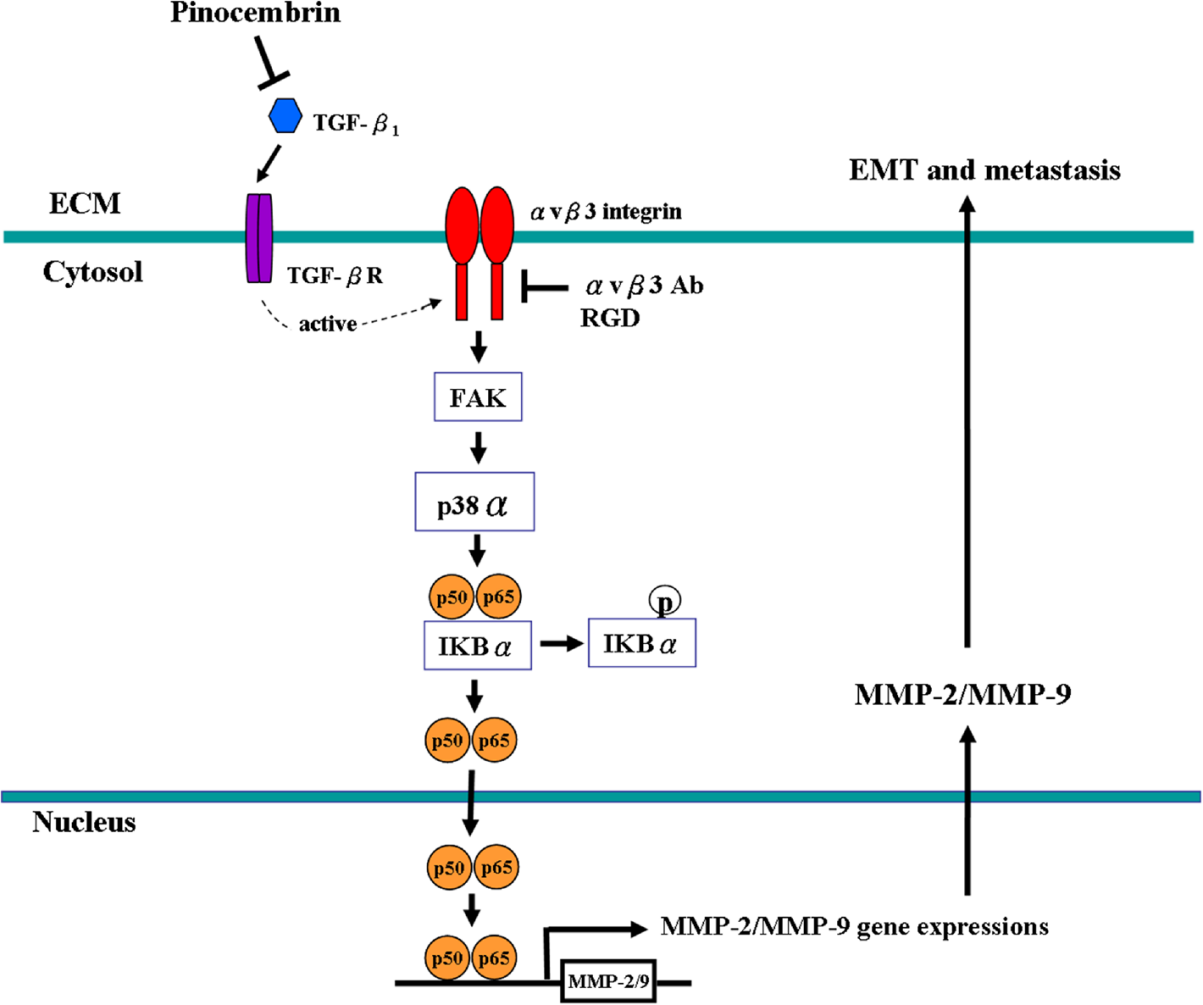
**Proposed mechanisms by which pinocembrin suppresses TGF-β1-induced epithelial-mesenchymal transition and metastasis in human Y-79 cells.** Pinocembrin inhibits TGF-β1-acted through avβ3 integrin receptor to activate FAK and p38α, leading to the phosphorylation of IκBα, activation of NF-κB (p50 and p65), nuclear translocation, NF-κB binding to MMP-2/MMP-9 promoter binding sites, initiation of MMP-2/MMP-9 gene expressions, and contributing to the inhibition of cell EMT and metastasis.

In view of these previous findings, we proposed a schematic presentation of possible mechanisms for the antimetastatic activity of pinocembrin could effectively inhibit TGF-β1-induced EMT and metastasis of Y-79 cells by decreasing MMP-2 and MMP-9 expressions through the αvβ3 integrin receptor and FAK/p38α/NF-κB signaling pathway (Figure 7). Based on our findings, we suggest that pinocembrin promotes a strong protective effect against TGF-β1-mediated metastasis via down-regulation of early and long-term inside-out signaling processes. The findings and concepts disclosed here provide an important basis for a further exploration of the action mechanisms of pinocembrin and its potentially beneficial effect in the prevention of tumor metastasis.

## Competing interests

The authors declare that they have no competing interests.

## Authors’ contributions

KSC, CSC and YWS designed the experiment. KSC and YWS performed the experiments. KSC, MDS and YWS analysed data and wrote the paper. All authors read and approved the final manuscript.
